# Genomic resources for two apex avian predators from Saudi Arabia: *Falco biarmicus* and *Falco peregrinus*

**DOI:** 10.1093/g3journal/jkag061

**Published:** 2026-04-06

**Authors:** Andrea Zuccolo, Nahed Mohammed, Abdulmajeed Fahad Alrefaei, Sara Mfarrej, Umair Toor, Luis F Rivera, Saule Mussurova, Arnab Pain, Fiona McCarthy, Abdulwahed Fahad Alrefaei, Rod A Wing

**Affiliations:** Center for Desert Agriculture, Biological and Environmental Sciences and Engineering Division, King Abdullah University of Science and Technology, Thuwal 23955-6900, Saudi Arabia; Manash Kozybayev North Kazakhstan University, International Campus, Petropavlovsk 150000, Kazakhstan; Crop Science Institute, Sant’Anna School of Advanced Studies, Piazza Martiri della Libertà 33, Pisa 56127, Italy; Center for Desert Agriculture, Biological and Environmental Sciences and Engineering Division, King Abdullah University of Science and Technology, Thuwal 23955-6900, Saudi Arabia; Department of Biology, Jamoum University College, Umm Al-Qura University, Makkah 24382, Saudi Arabia; Center for Desert Agriculture, Biological and Environmental Sciences and Engineering Division, King Abdullah University of Science and Technology, Thuwal 23955-6900, Saudi Arabia; Center for Desert Agriculture, Biological and Environmental Sciences and Engineering Division, King Abdullah University of Science and Technology, Thuwal 23955-6900, Saudi Arabia; Center for Desert Agriculture, Biological and Environmental Sciences and Engineering Division, King Abdullah University of Science and Technology, Thuwal 23955-6900, Saudi Arabia; Center for Desert Agriculture, Biological and Environmental Sciences and Engineering Division, King Abdullah University of Science and Technology, Thuwal 23955-6900, Saudi Arabia; Center of Agrocompetence, Manash Kozybayev North Kazakhstan University, Petropavlovsk 150000, Kazakhstan; Center for Desert Agriculture, Biological and Environmental Sciences and Engineering Division, King Abdullah University of Science and Technology, Thuwal 23955-6900, Saudi Arabia; Animal Comparative and Biomedical Sciences, University of Arizona, Tucson, AZ 85721, United States; Department of Zoology, College of Science, King Saud University, P.O. Box 2455, Riyadh 11451, Saudi Arabia; Center for Desert Agriculture, Biological and Environmental Sciences and Engineering Division, King Abdullah University of Science and Technology, Thuwal 23955-6900, Saudi Arabia; School of Plant Sciences, Arizona Genomics Institute, University of Arizona, Tucson, AZ 85721, United States

**Keywords:** falcon genomics, genome assembly, transposable elements, structural variants

## Abstract

Falcons (genus *Falco*) are a rapidly diversified bird clade, with 38 species evolving over the past ∼7.5 million years. Despite their ecological and evolutionary significance, high-quality genomic resources remain limited. Here, we present two chromosome-level genome assemblies for *Falco peregrinus* and *Falco biarmicus*, enabling detailed analyses of genome architecture, gene content, transposable elements (TEs), and structural variation. Using PacBio HiFi sequencing, we generated highly contiguous assemblies (N50: 60.76–77.83 Mb) with >97% BUSCO completeness. Comparative analyses with *Gallus gallus* and *Falco rusticolus* revealed strong synteny among *Falco* species, whereas extensive chromosomal rearrangements were observed in comparison with the more distantly related *Gallus gallus*. TEs account for 7.43–8.44% of the genomes, with LINEs (CR1) and DNA transposons (Mutator, CACTA) predominating. Contigs belonging to the W sex chromosome were identified based on their significantly higher TE content (30% or more) compared to autosomes and the Z chromosome. Gene prediction, informed by long-read RNA Iso-Seq, identified 18,638–19,858 genes per genome, aligning with prior Falcon annotations. Importantly, we recovered key immune and sensory gene families, including MHC class I/II, innate immune receptors, and 24–25 olfactory receptor genes.

We detected 8,746 structural variants, over 40% of which involved TEs, underscoring their role in genome polymorphism. These assemblies provide a valuable resource for investigating avian chromosome evolution, TE dynamics, and species-specific adaptations. They also establish a foundation for comparative genomics, population genetics, and conservation efforts in falcons.

## Introduction

Class Aves encompasses 70 million years of evolution and includes more than 10,000 living species (Jarvis et al. 2014; Prum et al. 2015), displaying extreme diversity in morphology, behavior, and ecology. Falcons belong to the clade Inopinaves, which includes 38 species (Wink 2018) arranged into four major groups based on their morphology and behaviors: Kestrels, Hierofalcons, Peregrine falcons, and Hobbies. The divergence and the diversification of falcons took place over ∼7.5 million years, a timescale comparable to that of early hominid evolution (Fuchs et al. 2015). This rapid and recent diversification provides valuable insights into the ecological and geographical factors that have shaped this diversification (Fuchs et al. 2015; Wilcox et al. 2019). Falcons have diversified through multiple radiations, resulting in approximately 38 extant species within the genus Falco, a level of species richness that exceeds the average number of species per avian genus (∼4–5 species), as calculated and reviewed by Wilcox et al. (2019) from IOC World Bird List data by Gill and Donsker (2019) Consequently, falcons represent an ideal group for studying speciation mechanisms across evolutionary time scales.

Most bird species have diploid karyotypes consisting of approximately 2*n* = 80 chromosomes, typically including 7 to 10 pairs of large and medium-sized macrochromosomes, one pair of sex chromosomes (W and Z), and a large number of morphologically similar microchromosomes (Masabanda et al. 2004). Interestingly, the karyotype of Falconinae (a subfamily within the Falconiformes) differs notably from the typical avian karyotype, displaying a lower chromosome count (2*n* = 40–52), limited size variation among macrochromosomes, and a reduced number of microchromosomes. The reduced chromosome number in falcons is primarily the result of microchromosome fusion events that gave rise to new macrochromosomes (Nishida et al. 2008).

Recent improvements in genome sequencing, including advanced assembly methods and long-range technologies such as optical mapping (Schwartz et al. 1993) and Hi-C chromatin interaction analysis (Lieberman-Aiden et al. 2009; Duan et al. 2010; Burton et al. 2013), have greatly enhanced the quality of genome assemblies (Sedlazeck et al. 2018). These high-resolution genomes serve as essential tools for in-depth research in the ecology, conservation, evolutionary biology, and population genetics of both wild and domesticated species (Whibley et al. 2021).

The peregrine falcon (*Falco peregrinus*) belongs to the peregrine falcon group. It is among the world's fastest flying animals, capable of achieving speeds over 320 km/h during its hunting stoop (Tucker 1998). Depending on the region and sex, peregrine falcons’ length ranges from 34 to 58 cm, and weight from 330 to 1,500 grams, with females being significantly larger than males (White et al. 2024). The peregrine falcon is highly migratory and can be found almost globally, from the Arctic tundra to the tropics, and is adaptable to environments, including urban areas, coastlines, and mountains (Cade et al. 1988; Del Hoyo et al. 1994). The lanner falcon (*Falco biarmicus*) is part of the Hierofalcons group. Its average length ranges from 40 to 50 cm, and it typically weighs between 500 and 800 grams. It is native to Africa, the Mediterranean, and parts of the Middle East, and prefers open landscapes such as savannas, semi-deserts, and cliffs. Unlike the peregrine falcon, the lanner falcon is less migratory, with most populations being sedentary or making only short seasonal movements (Ferguson-Lees and Christie 2001; White et al. 2024).

Building on the first genome assemblies provided for falcons by Zhan et al. (2013), genomic resources for falcons have expanded substantially over the past few years (particularly between 2023 and 2025) (Zuccolo et al. 2023; Morales et al. 2024; Al-Ajli et al. 2025). However, the incorporation of new high-quality, chromosome-level assemblies remains valuable for improving comparative and functional genomic analyses in this ecologically important and rapidly diversifying group. High fragmentation, as well as a lack of detailed annotation, are significant limitations affecting most existing genomic resources for Falconidae, hindering comparative and evolutionary analyses. To bridge this gap, we provide comprehensive genome assemblies and annotations for two representative species, *Falco biarmicus* and *Falco peregrinus*, enabling more detailed investigations of genomic architecture, structural variation, and adaptive evolution in falcons. In this study, we generated and annotated high-quality genome assemblies for two falcon species, including comprehensive annotation of genes and transposable elements. We assessed genome completeness using gene ontology analyses and focused on improving the annotation of gene families relevant to adaptation, such as immune-related genes and sensory receptors.

We further conducted comparative genomic analyses between the two falcon species and with other avian genomes to investigate chromosomal organization, structural variation, and genome evolution. These analyses provide a framework for exploring evolutionary processes, adaptive traits, and conservation-relevant genomic features in falcons, including immune system diversity and sensory adaptations.

## Materials and methods

### Sample collection and HMW DNA extraction

**Fig. 1.**
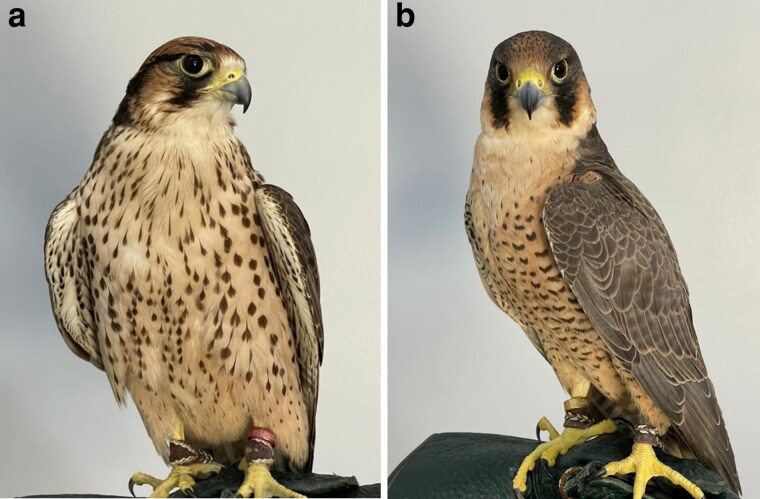
Adult female specimens of *Falco biarmicus* (a) and *Falco peregrinus* (b) used for genome sequencing and assembly.

High molecular weight (HMW) DNA was extracted from the blood samples of two adult female specimens of *Falco biarmicus* and *Falco peregrinus* (Fig. 1) using the Qiagen Genomic Tip 100/G Kit® (Qiagen, Germany), following the manufacturer's protocol. To minimize lysate viscosity and improve lysis efficiency, the blood was homogenized in phosphate-buffered saline (PBS). This step is particularly important for avian species due to their nucleated erythrocytes. DNA quantity was measured using a broad-range Qubit fluorometer (Invitrogen, USA), and fragment size distribution was evaluated using the Femto Pulse system (Agilent Technologies, USA) prior to library preparation.

### PacBio library construction and DNA sequencing

The extracted DNA was sheared into fragments of approximately 10–30 kb using the Megaruptor 3 system (Diagenode, USA). Library preparation followed the SMRTbell Prep Kit 3.0 protocol, with final size selection between 10–25 kb using the PippinHT electrophoresis system (Sage Science, MA, USA). Libraries were quantified with the Qubit High-Sensitivity assay (Invitrogen), and fragment sizes were re-confirmed using the Femto Pulse system. For sequencing, libraries were bound using the PacBio Sequel II Binding Kit 3.2 and sequenced on the PacBio Sequel II platform with 8 M SMRT cells in Circular Consensus Sequencing (CCS) mode with the Sequel II Sequencing Kit 2.0. Sequencing runs were carried out at the KAUST Bioscience Core Labs with 30-hour durations.

### RNA extraction and iso-seq sequencing

RNA from *F. peregrinus* and *F. biarmicus* was extracted from blood using the Zymo-Direct Zol kit (Zymobiomics) with DNase I treatment. The extraction protocol was optimized by extending the TRIzol lysis incubation and adding chloroform during the initial lysis step to ensure a clear aqueous RNA phase. RNA quality was assessed using the Qubit Broad Range kit (Invitrogen) and the RNA 6000 Nano LabChip kit (Agilent), yielding an RNA integrity number (RIN) of 9. Iso-Seq libraries were prepared following the PacBio protocol—PN 102-396-000 REV02 APR2022. Library construction began with 300 ng of total RNA for cDNA synthesis using the NEBNext® Single Cell/Low Input cDNA Synthesis & Amplification Module, followed by cDNA repair, A-tailing, adapter ligation, nuclease treatment, and bead cleanup with PacBio SMRTbell Prep Kit 3.0. Libraries were quantified with a Qubit Fluorometer and assessed for fragment size using an Agilent Bioanalyzer with the High Sensitivity DNA Kit. Sequencing was performed using the PacBio Revio polymerase kit and Revio sequencing plate in CCS mode on 25 M SMRT Cells (one per species) for 24 h on the PacBio Revio system.

### Genome assembly, quality assessment, and validation

HiFi reads were used for genome assembly with HiFiAsm v0.19.8 (Cheng et al. 2024) under default settings. Assembly metrics were calculated using QUAST/v5.2.0 (Gurevich et al. 2013). Assembly completeness was assessed with BUSCO, v5.7.1 (Simão et al. 2015) using the aves gene database, aves_odb10, which includes 8,338 genes. Genome assembly quality was assessed using Merqury (Rhie et al. 2020) to estimate base-level consensus accuracy and k-mer completeness.

### Transposable element identification and annotation

To generate a transposable element (TE) library for the two genome assemblies, we used the tool EDTA/v2.1.0 (Ou et al. 2019). The resulting libraries were then used with Repeat Masker/v4.1.4 (Smit et al. 2015), run under default settings, to quantify TE content and to softmask TEs in the genome assemblies prior to the gene prediction step.

### Gene prediction

AUGUSTUS (Stanke and Waack 2003) was used to perform gene prediction, employing the gene models derived from *G. gallus*. Iso-seq data were used as extrinsic support. These specific parameters were used: –sample=200 –alternatives-from-sampling=false –maxtracks=1. Gene structure statistics were calculated using the AGAT tool (Dainat 2024).

### Functional annotation and analysis

Proteomes generated by AUGUSTUS were functionally annotated using InterProScan ver 5.75-106 (Jones et al. 2014) to obtain conserved motifs, gene ontology, and biological pathways information. Functional annotations were compared to those provided by NCBI for *F. peregrinus* (GCF_023634155.1), *F. biarmicus* (GCF_023638135.1), and *F. rusticolus* (GCF_015220075.1) reference genomes, including associated Gene Ontology provided by NCBI. The current NCBI reference assemblies for these species were chosen because, as reference assemblies, they are representative of an accessible, high-quality annotation used as a community benchmark for genome improvements and updates. Gene Ontology (GO) annotations from each Gene Ontology Annotation File (GAF) were summarized using the Generic GO Slim set (Gene Ontology Consortium 2021). For each dataset, gene products were assigned to GO Slim categories, and the relative frequency of each category was calculated as a percentage of the total annotated gene products. These distributions were then compared across datasets. Manual review of InterPro motifs was used to identify and compare key gene sets that are often poorly annotated in birds or of interest to conservation and diversity studies.

### Interspecies comparison and structural variants detection

The genome assemblies of *F. peregrinus* and *F. biarmicus* were compared with those of the gyrfalcon (*F. rusticulus*) (GenBank assembly accession JAPSEQ000000000) and *G. gallus* (GenBank assembly accession: GCA_000002315.5) using Mash map (Jain et al. 2018) and D-Genies (Cabanettes and Klopp 2018).

To identify structural variants, the *F. biarmicus* genome assembly was used as a reference guide, and *F. peregrinus* Pac Bio HiFi reads were mapped onto it using minimap2/v2.24 2 (Li 2018) with default parameters. The output SAM file was then indexed and sorted using samtools/v1.16.1 (Danecek et al. 2021). Structural variants (SVs), including insertions, inversions, deletions, duplications, interspersed duplications, and tandem duplications, were detected by processing the output file with the SVIM-asm tool (Heller and Vingron 2020).

### Phylogenetic analyses

Multiple sequence alignments for the *Falco* spp. contigs homologous to *G. gallus* chromosome 1, as well as the corresponding Neighbor-Joining tree, were generated using the software package Hakmer (Sanderson et al. 2017) to further assess orthology and assembly consistency among homologous regions.

## Results and discussion

### Genome sequencing and assembly

Approximately 175 Gbp and 160 Gbp of HiFi PacBio reads were generated for *F. peregrinus* and *F. biarmicus* genomes, respectively. The reads N50 parameter was greater than 20 Kbp for both assemblies. The total amount of sequence data provided coverages of 125 X for *F. peregrinus* and 114 X for *F. biarmicus*, assuming a genome size estimate of 1.4 Gbp for both species. The genome size estimates for *F. biarmicus* and *F. peregrinus* were obtained from www.genomeark.org (Rhie et al. 2021). The final assembly of *F. peregrinus* (FPA) consists of 849 contigs with a total length of ∼1,370 Gbp, which is slightly shorter than that of *F.biarmicus* (FBA), which was composed of 709 contigs, having a total length of ∼1.377 Gbp. Both genome assembly lengths are in agreement with the estimated genome sizes for the respective species (Rhie et al. 2021). The N50 was 60.76 Mbp for FPA and 77.83 Mbp for FBA (Table 1).

**Table 1.** Assembly statistics for the *Falco biarmicus* and *Falco peregrinus* genome assemblies generated using PacBio HiFi sequencing.

	*Falco biarmicus*	*Falco peregrinus*
# Contigs	709	849
# Contigs > 50,000 bp	381	440
Total length (bp)	1,377,200,195	1,370,524,198
Largest contig length (bp)	125,314,217	123,605,428
GC (%)	43.52	42.87
N50	77,826,137	60,761,372
N75	33,957,898	24,984,432
L50	7	9
L75	14	17

There are three publicly available genome assemblies for *F. biarmicus* (two of which provide primary and alternate haplotypes) and six for *F. peregrinus* (four of which provide primary and alternate haplotypes). The statistics for each of these assemblies are provided in Supplementary Table 1.

Two and three genome assemblies were generated using Illumina technology for *F. biarmicus* and *F. peregrinus*, respectively. As expected, for all comparisons, the contiguity of our assemblies is substantially higher than that of the Illumina assemblies, as indicated by the lower number of contigs and the correspondingly improved N50 and L50 statistics.

In the case of the Vertebrate Genomes Project (VGP) assemblies (Rhie et al. 2021), which were generated using a combination of technologies with a PacBio backbone, assembly metrics are often comparable at the scaffold level (with slightly greater contiguity for the VGP assemblies) whereas at the contig level our assemblies perform better reflecting the higher coverage of PacBio HiFi reads used in this study. Overall, our assemblies represent a clear improvement over the Illumina-based assemblies and compare favorably with the VGP assemblies, effectively complementing them.

### Evaluation of genome assembly quality

We evaluated the completeness of both genome assemblies using BUSCO analysis (Supplementary Table 2) with the aves_odb10 dataset, which includes 8,338 genes (Simão et al. 2015). The BUSCO scores were 97.3% for FBA and 97.2% for FPA. The number of missing genes was 184 and 189 for FBA and FPA, respectively, amounting to ∼2.3% of the dataset. Notably, these BUSCO scores were slightly better than those reported for the genome assembly of the gyrfalcon (*Falco rusticolus*), which had 96.7% completeness and 210 missing BUSCOs (Zuccolo et al. 2023). To further assess assembly quality, we evaluated base-level accuracy and k-mer completeness using Merqury (Supplementary Table 3). Both genome assemblies exhibited very high consensus accuracy, with QV values exceeding 60, corresponding to estimated base-level error rates below 1 × 10^−6^. K-mer completeness was also high for both assemblies, indicating that the majority of read-derived sequence content is well represented. Although minor differences were observed between assemblies, both genome assemblies are of very high quality and suitable for downstream genomic analyses.

### Chromosome assignment for *Falco peregrinus* and *Falco biarmicus* genome assemblies

Chromosome assignment was performed for the FBA and FPA by comparing their largest contigs (those longer than 5 Mbp) to the reference genomes of chicken (*Gallus gallus*) and gyrfalcon (*F. rusticolus*) using dot plot analyses (dot plots are available in the Data availability section). For *F. biarmicus*, 27 out of 28 large contigs showed clear homology with at least one chromosome from either *G. gallus* and/or *F. rusticolus*. Among these, six contigs displayed canonical telomeric repeats (TTAGGG) at both ends, 18 at one end, and three lacked telomeric sequences entirely (Table 2). When aligned to the *G. gallus* genome, eighteen contigs each showed a single alignment match to a *G. gallus* chromosome; whereas eight contigs showed matches to two or more *G. gallus* chromosomes; and one contig did not have a convincing alignment match to the *G. gallus* genome (Table 2). Against the *F. rusticolus* genome, eight contigs matched to two or more scaffolds or contigs, while the remaining 19 each aligned to a single scaffold or contig (Table 2). For *F. peregrinus*, 30 of 39 contigs longer than 5 Mbp exhibited homology with the *G. gallus* and/or *F. rusticolus reference genomes*. Four contigs contained telomeric repeats at both ends, 19 at one end, and seven lacked telomeric sequences (Table 3). Nine contigs aligned to two or more *G. gallus* chromosomes (Table 3), and two contigs did not show significant alignment matches when compared to the *G. gallus* genome assembly. When compared to the *F. rusticolus* genome assembly, all the contigs showed similarity; thirteen aligned with two or more scaffolds or contigs, while the rest had alignment matches to a single scaffold or contig (Table 3). The nine contigs of *F. peregrinus* and the single contig of *F. biarmicus*, out of those longer than 5 Mbp, that did not show significant similarity to either the *G. gallus* or *F. rusticolus* reference genomes may represent highly diverged, highly heterozygous, or structurally rearranged genomic regions; all but one of these contigs are highly repetitive and enriched in TEs.

**Table 2.** Characteristics of *Falco biarmicus* genome assembly contigs larger than 5 mb, including contig length, GC content, predicted gene number, transposable element (TE) content, telomeric features, and homology to *Gallus gallus* (GenBank assembly accession: GCA_000002315.5) and *Falco rusticolus* chromosomes (GenBank Assembly accession JAPSEQ000000000). Contigs marked with “*” are considered putative tracts of chromosome W due to their high TE content.

Contig name	Length (bp)	GC (%)	Number of predicted genes	TE content (%)	Homologous sequence(s) in *F. rusticulus*	Homologous chromosome in *G. gallus*	Telomeric like ends
ptg000007l	125,314,217	40.49	1,068	10.2	1_sc	1	2
ptg000010l	115,284,532	41.96	1,566	6.68	4_sc, 14co	19, 4, 15	1
ptg000013l	107,198,156	40.67	813	10.7	3_sc	2	1
ptg000001l	104,188,287	43.08	1,666	4.66	2_sc, 23_sc	12, 28, 14, 2	1
ptg000002l	93,090,950	40.78	850	8.45	5_sc	3	2
ptg000009l	88,285,053	40.58	861	10.33	9_sc, 16_sc	Z	2
ptg000003l	77,826,137	41.81	1,026	5.86	6_co	1	1
ptg000011l	75,311,914	42.53	1,056	7.17	7_sc	10, 5	2
ptg000008l	58,798,505	43.72	862	8.95	10_sc	17, 6	2
ptg000004l	54,366,635	42.54	742	3.55	8_sc	7, 13	1
ptg000033l	40,195,000	44.37	782	5.4	11_sc	20, 5	1
ptg000026l	39,106,859	42.95	587	9.86	12_co	8	1
ptg000017l	36,370,341	42.73	525	7.1	13_sc	3	2
ptg000029l	33,957,898	43.83	474	14.38	15_co	9	1
ptg000005l	25,921,067	43.34	463	9.51	18_sc	11	1
ptg000151l	24,691,839	43.83	350	4.86	14_co	4	1
ptg000034l	17,769,550	41.3	129	22.23	20_co	1	1
ptg000020l	17,750,605	48.59	536	2.87	19_sc	21, 23	1
ptg000012l	14,911,588	50.76	455	11.77	27_c0, 30_co	26	1
ptg000015l	14,462,765	46.86	309	2.81	14_co	18, 19	1
ptg000006l*	13,336,256	45.7	76	70.84	21_sc, 23_sc	?	1
ptg000050l	12,635,417	43.73	143	13.87	8_sc	7	–
ptg000027l	10,439,594	42.37	111	16.57	2_sc	2	1
ptg000023l	10,283,805	49.17	246	10.07	22_co	24	1
ptg000021l*	9,983,502	42.01	45	38.29	29_sc, 26_sc, 21_sc	Z	–
ptg000045l*	9,858,160	43.11	94	36.79	26_sc, 31_co, 32_co	Z	–
ptg000057l	6,192,117	48.3	126	20.5	25_sc, 11_sc	22	1

**Table 3.** Characteristics of *Falco peregrinus* genome assembly contigs larger than 5 mb, including contig length, GC content, predicted gene number, transposable element (TE) content, telomeric features, and homology to *Gallus gallus* (GenBank assembly accession: GCA_000002315.5) and *Falco rusticolus* chromosomes (GenBank assembly accession JAPSEQ000000000). Contigs marked with “*” are considered putative tracts of chromosome W due to their high TE content.

Contig name	Length (bp)	GC (%)	Number of predicted genes	TE content (%)	Homologous sequence(s) in *F. rusticulus*	Homologous chromosome in *G. gallus*	Telomeric like ends
ptg000003l	123,605,428	40.35	1,100	8.17	1_sc	1	2
ptg000002l	113,689,277	41.8	1,613	5.08	4_sc, 14_co	4, 15, 19	1
ptg000017l	105,006,225	40.44	812	7.75	3_sc	2	–
ptg000012l	77,782,607	41.78	1,061	5.81	6_co	1	1
ptg000014l	70,045,625	42.2	1,078	4.54	7_sc	5, 10	1
ptg000022l	68,295,893	40.41	663	9.06	9_sc, 16_sc	Z	–
ptg000005l	65,889,360	42.65	921	3.64	8_sc	7, 13	2
ptg000016l	61,626,678	45.1	1,366	4.41	2_sc, 23_sc	2, 12, 14, 28	1
ptg000025l	60,761,372	41.33	645	5.77	5_sc	3	1
ptg000006l	54,965,574	43.35	900	4.43	10_sc	6, 17	1
ptg000008l	52,714,140	40.53	515	5.02	2_sc	2	1
ptg000011l	38,891,253	44.04	763	4.27	8_sc, 11­­_sc	5, 20	2
ptg000019l	36,813,361	42.42	582	4.30	8_sc, 12_co	8	2
ptg000018l	34,186,737	42.26	497	3.36	13_sc	3	–
ptg000001l	29,990,544	42.94	467	3.31	15_co	9	1
ptg000009l	28,483,116	42.63	465	11.24	5_sc, 18_sc	11	1
ptg000020l	24,984,432	44.02	467	4.98	17_sc	4	1
ptg000013l	19,621,702	42.72	195	10.96	16_sc	Z	–
ptg000030l	17,686,102	48.5	526	2.84	19_sc	21, 23	1
ptg000021l	17,269,478	39.41	115	8.11	5_sc	3	1
ptg000031l	14,425,801	46.83	350	3.09	14_co	18, 19	1
ptg000050l	13,747,739	39.16	90	8.71	5_sc	3	–
ptg000032l	13,662,493	39.36	98	8.13	20_co	1	–
ptg000245l	13,105,404	45.65	52	21.62	6_co, 21_sc	16	1
ptg000075l	12,809,879	49.22	341	4.64	21_sc, 25_sc	1, 3, 22	1
ptg000027l	12,777,616	51.16	477	7.03	27_co, 30_co	26	1
ptg000034l*	11,509,719	43.59	153	36.72	26_co, 31_co, 32_co	?	–
ptg000004l*	11,020,315	46.09	742	33.60	21_sc, 26_sc	Z	1
ptg000043l*	10,600,803	44.91	183	45.26	7_sc, 21_sc, 28_co	?	1
ptg000038l	10,119,700	49.58	354	4.23	8_sc, 22_co	24	1

### Chromosome rearrangements

**Fig. 2.**
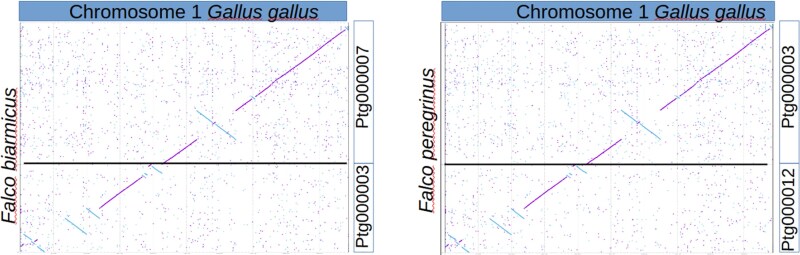
Dot plot comparison of *Gallus gallus* chromosome 1 (*x*-axis) vs *Falco biarmicus* and *Falco peregrinus* contigs (*y*-axis), showing conserved chromosomal synteny.

Multiple contig alignments to different reference chromosomes suggest the presence of potential chromosome fusions or breakages. An illustrative example is provided by *G. gallus* chromosome 1, which is covered by two separate contigs in both FBA (ptg000007, ptg000003) and FPA (ptg000012, ptg000003) (Fig. 2). Further comparison with the *F. rusticolus* genome assembly (Supplementary Fig. 1) revealed the same pattern, indicating that this arrangement is conserved across at least three *Falco* species. Notably, FBA contig ptg000007 and FPA contig ptg000003 display telomeric repeats at both ends, suggesting that they may represent complete chromosomes, whereas the other two contigs have telomeric repeats at only one end. The genome assemblies of three different *Falco* species, obtained independently, support this chromosomal arrangement, although the lack of Hi-C or other independent evidence for FBA and FPA does not completely rule out the possibility that these observations are artifactual. Notably, one of the assemblies (*F. rusticulus*) is also supported by optical mapping data. Given the older evolutionary origin of the genus *Gallus* compared to *Falco*, this suggests that the pattern observed in *Falco* species likely resulted from a fission event that split chromosome 1 into two. This evidence is consistent with the reported higher rate of chromosomal rearrangements in *Falco* species (Nishida et al. 2008), especially when compared to the generally slow rate of chromosomal evolution observed in most other birds (Ellegren 2010).

Future incorporation of Hi-C or other long-range chromatin conformation data will be necessary to definitively validate chromosome-scale structures and to distinguish true rearrangements from potential assembly artifacts.

### *Falco spp*. interspecific comparison

**Fig. 3.**
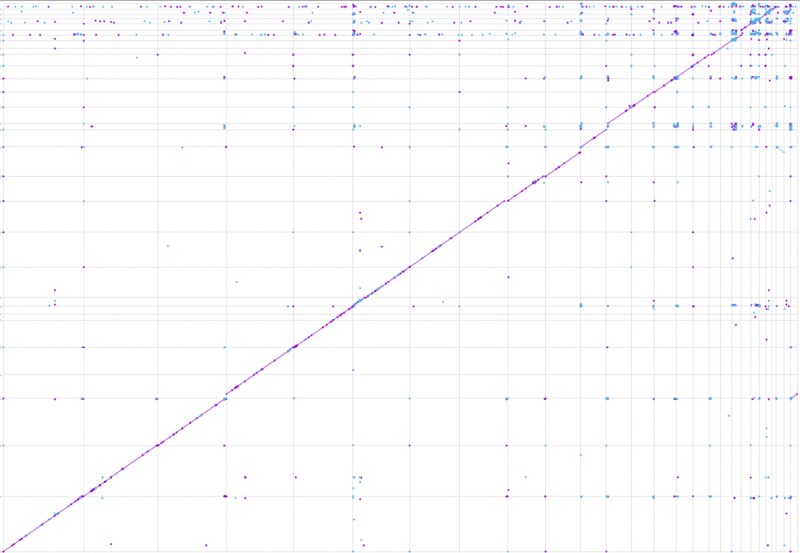
Whole-genome dot plot comparison between *Falco biarmicus* (*x*-axis) and *Falco peregrinus* (*y*-axis), showing high chromosome-level collinearity and the absence of large-scale rearrangements between the two species.

FBA and FPA were compared to each other (Fig. 3), as well as to the genome assemblies of *F. rusticolus* and *G. gallus* using dot plot analyses (Supplementary Fig. 2). All three *Falco* species show a high level of collinearity at the chromosome level, with no large rearrangements visible. This confirms the close evolutionary relationships among the three species and suggests a high level of conservation in their chromosome structure. As expected, comparison of the *Falco* spp. genome assemblies with the *G. gallus* genome assembly reflect the greater evolutionary distance between the species, with several rearrangements, including inversions and translocations, along with a marked drop in sequence similarity. To further verify orthology and assembly consistency at the sequence level, we aligned the largest contigs homologous to *G. gallus* chromosome 1 from the three *Falco* species and inferred a phylogenetic tree (Supplementary Fig. 3), using the homologous *G. gallus* chromosome as an outgroup. Recovery of the expected species relationships, with *F. biarmicus* clustering with *F. rusticolus*, supports the correct assembly and orthologous assignment of these homologous chromosomal regions.

### Transposable element identification and characterization

Two specific TE libraries were created by running the EDTA tool (Ou et al. 2019) on each genome assembly. They contain 5,199 and 5,410 entries for FBA and FPA, respectively (Supplementary Table 4). The proportion of the genome identified as TE-related sequence was 7.43 and 8.44% for FBA and FPA, respectively (Supplementary Table 5). These values are largely consistent with those identified in other birds and confirm the underrepresentation of TEs in these genomes as compared to other metazoan assemblies (Chalopin et al. 2015; Kapusta and Suh 2017; Sotero-Caio et al. 2017). However, they are higher than many previous estimates for *Falco* spp.; for example, for both saker and peregrine falcon, the repetitive fraction (not limited to TEs) was estimated to be about 6.8% (Zhan et al. 2013). A TE-specific assessment for *F. peregrinus* set the amount of these sequences at 5.5% (Zhang et al. 2014), whereas another study of eight different falcon species found an average TE content of 6.5% (Wilcox et al. 2022). The TE content for *F. rusticolus* was 7.61% (Zuccolo et al. 2023). In both FBA and FPA, the most abundant TE is the element CR1, at 3.30 and 3.35%, respectively. CR1 is a LINE TE widely distributed across vertebrates but is more abundant in birds, reptiles, and some fish (Kapitonov and Jurka 2003; Sotero-Caio et al. 2017). It was first identified in *G. gallus*, where it represents at least 6.4% of that genome (International Chicken Genome Sequencing Consortium 2004). One possible explanation for the lower amount of CR1 in *Falco* spp. is that these genomes may harbor a higher proportion of ancient, degraded copies of this TE family. Such copies could remain undetected by standard TE annotation methods due to extensive sequence divergence (Hoen et al. 2015; Platt et al. 2016). Other abundant TE families include the DNA-TE CACTA (1.62 and 1.26% in FBA and FPA, respectively) and the class 1 LTR-RT (1.81 and 2.08% in FBA and FPA, respectively). Notably, the DNA-TE Mutator elements are almost four times more abundant in FPA than in FBA (1.65 and 0.42%, respectively), accounting for much of the difference in the overall TE content between the two genomes. In FPA, these elements are significantly more abundant than in *F. rusticolus*, where they account for just 0.54% of the genome (Zuccolo et al. 2023). In both FBA and FPA, TE content varies widely between different contigs. When considering only contigs larger than 5 Mbp, TE content ranges from 2.81 to 70.84% in FBA and from 2.84 to 45.26% in FPA (Tables 2 and 3).

TE content was used to aid in the correct assignment of genome assembly contigs showing similarity to the sex-determining W chromosome, taking advantage of its distinctive feature, i.e. its high transposable element (TE) content (Peona et al. 2021), which can serve as a diagnostic biomarker. We therefore considered contigs with significantly higher TE content (i.e. greater than 30% above the genome-wide average) as likely representative fragments of the W chromosome. Three contigs in FBA (ptg000006, ptg000021, ptg000045) and three in FPA (ptg000034, ptg000004, ptg000043) met this criterion and may represent segments of the W sex chromosome (Tables 2 and 3). Three of these contigs (ptg000021 and ptg000045 in FBA, and ptg000004 in FPA) showed homology to the Z chromosome of *G. gallus*. We note, however, that in this specific case, Z chromosome homology does not necessarily imply Z linkage. Given the extreme repeat enrichment of the avian W chromosome, sequence similarity is expected to be detectable primarily in repeat-poor regions corresponding to ancestral Z-W homologous sequences, leading to preferential alignment to the chicken Z chromosome. Accordingly, these contigs are considered likely to represent putative regions of the W chromosome.

### Gene prediction

The two genome assemblies, appropriately masked for TEs, were then analysed for gene content using the gene prediction software AUGUSTUS (Stanke and Waack 2003) using gene models from *G. gallus*. As a source of extrinsic supporting data, PacBio IsoSeq reads were employed: 881,978 reads and 4,249,326 reads for FBA and FPA, respectively. In total, 18,638 genes (including 2,269 monoexonic) and 19,858 genes (including 2,321 monoexonic) were predicted for the FBA and FPA assemblies, respectively. These values align well with those calculated in *F. rusticolus* (19,602 genes; Zuccolo et al. 2023) and are consistent with the number of predicted genes in many bird genomes (Zhang et al. 2014). However, they are higher than those previously obtained for *F. peregrinus* (16,263 genes) and for *F. cherrug* (16,204 genes) (Zhan et al. 2013). When predictions with an Augustus posterior prediction probability lower than 0.3 are excluded, the resulting gene counts (14,208 for FBA and 15,631 for FPA) appear to be in good agreement with these earlier estimates. In addition, gene annotation completeness was evaluated using BUSCO in protein mode based on the predicted protein sets generated by AUGUSTUS with Iso-Seq transcript support. BUSCO analysis recovered 80.3% (Single copy: 79.2%, Duplicated: 1.2%) and 83.2% (Single copy: 81.6%, Duplicated: 1.5%) complete orthologs for *F. biarmicus* and *F. peregrinus*, respectively. These values are lower than the corresponding genome-mode completeness scores (97.3 and 97.2%), indicating that a subset of conserved loci present in the assemblies was not recovered as valid coding sequences in the predicted gene set and are therefore absent from the predicted proteome used for protein-mode BUSCO assessment. This discrepancy likely reflects incomplete transcriptome representation and the conservative gene model inference inherent to AUGUSTUS under default parameter settings, despite the incorporation of Iso-Seq support, rather than true gene loss. The low duplication rates observed in protein mode are consistent with this conservative prediction behavior and may partially explain differences in predicted gene number relative to previous annotations in related falcon species.

For *F. biarmicus,* the mean gene length was 20,722 bp, with a median of 9,485 bp and an average of 8.7 exons per gene. The overall number of introns was 143,094, with an average length of 2,495 bp. For *F. peregrinus,* the mean gene length was 21,574 bp, with a median of 9,424 bp average of 9.1 exons per gene. The overall number of introns was 161,814, with an average length of 2,479 bp. All these values are highly consistent with the gene predictions for *F. rusticolus* (Zuccolo et al. 2023).

### Comparing genes function across falcon genomes

Gene ontology terms were assigned to 14,272 (76.57%) of *F. biarmicus* and 14,647 (73.76%) of *F. peregrinus* proteins. Comparison of Gene Ontology annotation for proteomes reported in this study with public reference genomes (see Materials and Methods) for the same species indicated that the relative assignment of GO categories differed by less than 2% for each functional class in both *F. biarmicus* and *F. peregrinus*. This finding indicates that these proteomes have a broadly comparable functional composition and is consistent with the high BUSCO scores obtained for these genome assemblies and gene identification. However, this summary does not indicate the presence of specific gene sets routinely studied for gene conservation and diversity of falcon populations, nor does the BUSCO assessment of highly conserved single copy genes indicate the utility of the genome for these studies.

To investigate our gene annotations in greater detail, we analysed both the Major Histocompatibility Complex (MHC) and the olfactory receptor genes (OR genes). The Major Histocompatibility Complex (MHC) plays a crucial role in population genetics due to its high polymorphism and its influence on immune responses and mate selection. Annotation of the two new falcon genomes revealed four MHC-related genes in each of the two assemblies. These include two and three annotated MHC class I and II genes in FBA and FPA, respectively (Table 4), which until now have been classified as low-quality or incomplete annotations in the reference assemblies specified in the Materials and Methods section.

**Table 4.** Comparison of immune-related gene families identified in the *Falco biarmicus* and *Falco peregrinus* genome assemblies generated in this study versus existing reference genome annotations, including MHC class I and II genes, Toll-like receptors, TRIM proteins, and natural killer cell receptors. Key immunological genes and their proteins were identified based on motif analysis and BLAST searches against the corresponding *G. gallus* proteins. For the reference genomes, NCBI protein accessions are reported, while the protein sequences for the genomes in this study are provided as supporting data.

Immune Markers	*F. biarmicus*		*F. peregrinus*	
	Reference	This Study	Reference	This Study
MHC class I	not found	2 (g1560.t1, g17983.t1^a^)	5^b^ (AFL93461.1^a^, AFL93460.1^a^, AFL93459.1^a^, AFL93458.1^a^, AFL93453.1^a^)	2 (g19173.t1, g10465.t1)
MHC class II alpha chain	not found	1 (g18179.t1)	not found	1 (g19474.t1^a^)
MHC class II beta chain	2 (XP_056181963^a^, XP_056183688^a^)	1 (g18178.t1^a^)	1 (XP_055658841^c^)	1 (g19473.t1)
Toll-like receptors	not found	2 (g14714.t1, g6488.t1)	1 (XP_055648394^c^)	3 (g1910.t1, g16914.t1, g4412.t1)
E3 ubiquitin-protein ligase TRIM7	1 (XP_056178936)	30 (see supporting data)	not found	20 (see supporting data)
Natural Killer cell receptors	2 (XP_056196515.1, XP_056179672.1)	11 (see supporting data)	10 (XP_055648525.1, XP_005234488.1, XP_055664100.1, XP_055664621.1, XP_027638718.1, XP_055647669.1, XP_027638256.1, XP_027638254.1, XP_055649153.1, XP_013152474.2)	12 (see supporting data)

^a^Partial sequences.

^b^Not mapped to the genome.

^c^Annotated as low-quality.

While the total number of annotated MHC genes remains limited, their presence as full gene models in these assemblies provides additional annotation consistency for this gene family.

Likewise, these genomes enable the identification of other innate immune receptors such as Toll-like receptors, natural killer cell receptors, and TRIM (Tripartite Motif) genes (Table 4). The annotations for these loci are consistent with those reported in existing assemblies and benefit from the overall contiguity and annotation framework of the present genomes. Table 4 includes representative reference and alternate assemblies commonly used for annotation comparison in these species; while additional assemblies exist, many are highly fragmented or lack comparable gene annotation, limiting their suitability for direct comparison.

A previous study investigating falcon-specific adaptations for predation noted that the peregrine falcon contained only 28 functional olfactory receptor genes (Zhan et al. 2013), despite earlier studies that demonstrated a positive correlation between olfactory bulb size and functional olfactory receptor genes (Steiger et al. 2008). Moreover, these results did not show the same olfactory receptor gene expansion reported in other birds (Steiger et al. 2009), a finding attributed to the reliance of falcons on the visual location of prey. Analysis of the new genome assemblies in this study identified 24 olfactory receptor genes in *F. biarmicus* and 25 olfactory receptor genes in *F. peregrinus*, which is in line with the initial 28 olfactory receptor genes reported.

### Structural variants

To evaluate the structural variation differences between the FBA and FPA genomes, we mapped the FPA PacBio reads onto the FBA genome assembly and assessed the SV difference using the SVIM-asm tool (Heller and Vingron 2020). In total, we identified 8,746 insertions and deletions. Of their entire sequence, 41.07% could be confidently associated with known TEs. Another 2.70% consisted of microsatellites and other low complexity regions. Notably, the TE content in insertions and deletions was significantly greater than the average for both host genomes (7.43 and 8.44% for FBA and FPA, respectively). This provides additional support for the important role of TEs as powerful generators of genomic variability (Kazazian 2004). Among the different classes of TEs, Long Terminal Repeats retroelements represented the majority of SV sequence length (35.16%), as well as SV hits: 5,731 out of 8,746 (65.5%) (Supplementary Table 5). LTR-RTs appear to be the major drivers of TE-related genome variation in the *Falco* species considered. Furthermore, 27 inversions were identified, the longest being 74,950 bp.

## Conclusions

We provided two contiguous high-quality genome assemblies for *F. biarmicus* and *F. peregrinus*, each with a length close to their estimated genome sizes. For both genomes, most of the large and medium-sized chromosomes were confidently assigned to their homologs in *G. gallus*. Interspecies comparisons, including both *F. rusticolus* and *G. gallus,* revealed evidence of breakages, fusions, as well as highly conserved regions. Furthermore, we identified 8,746 SVs when *F. biarmicus* and *F. peregrinus* were compared to each other. More than 40% of insertions and deletions were associated with TE activity, showing a marked enrichment of these elements in SVs compared to the average genome content (7.4 to 8.4%). TE content in *Falco* spp. The genomes are comparable to those of many other bird genomes. In addition, we identified about 19,000 genes per genome, which matches what has been found in other bird species. Importantly, our annotation recovered MHC class I and II genes, as well as other genes related to the immune system, innate receptors, and a more detailed set of olfactory receptor genes. This gives us a clearer picture of gene families important for immune function and sensory adaptation. Altogether, these resources offer a strong foundation for future studies on falcon genome biology, adaptive evolution, and conservation genomics.

## Supplementary Material

jkag061_Supplementary_Data

## Data Availability

All sequence data (Pac Bio HiFi long reads and Isoseq reads) are available at NCBI under the Bioproject accessions PRJNA1281327 and PRJNA1281361 for *F. biarmicus* and *F. peregrinus*, respectively. The sequence genome assemblies are publicly available at NCBI under accessions JBPRBC000000000 and JBPRBB000000000 for FBA and FPA, respectively. For each genome assembly, the gene annotation files, the dotplot comparisons of each contig against *G. gallus* and *F. rusticolus*, the TE libraries, GO annotation files, sequences used for olfactory receptor analysis and related BLAST results, and immune marker sequences were deposited in Zenodo under DOI: 10.5281/zenodo.15861596. Supplemental material available at G3 online.
