# Supplementary material for: Genomic resources for two apex avian predators from Saudi Arabia: Falco biarmicus and Falco peregrinus
Source: G3 (Bethesda). 2026 Apr 6;16(5):jkag061. doi: 10.1093/g3journal/jkag061 (PMC13148383; doi:10.1093/g3journal/jkag061)
Supplement: jkag061_Supplementary_Data [file jkag061_supplementary_data.docx]

|  | Technology | Chromosome  level? | Genome  coverage | Genome  size | Total  ungapped  length | Scaffolds | Contigs | Scaffold  N50 | Scaffold  L50 | Contig  N50 | Contig  L50 | Link |  |
| --- | --- | --- | --- | --- | --- | --- | --- | --- | --- | --- | --- | --- | --- |
|  |  |  |  |  |  |  |  |  |  |  |  |  |  |
| *F. biarmicus* | **PacBio HiFi** | **NO, contig** | **114 X** | **1.4 Gb** | **1.4 Gb** | **709** | **709** | **77.8** | **7** | **77.8** | **7** | **www.ncbi.nlm.nih.gov/datasets/genome/GCA_052915945.1/** |  |
|  | PacBio HiFi,  HiC, Bionano DLS | YES | 28 X | 1.3 Gb | 1.3 Gb | 397 | 555 | 91.5 | 6 | 24.9 | 13 | www.ncbi.nlm.nih.gov/datasets/genome/GCF_023638135.1/ | PRI |
|  | PacBio HiFi,  HiC, Bionano DLS | NO, contig | 28 X | 1.2 Gb | 1.2 Gb | 3090 | 3090 | 1.8 | 188 | 1.8 | 188 | www.ncbi.nlm.nih.gov/datasets/genome/GCA_023634305.1/ | ALT |
|  | Illumina | NO, scaffold | 39.22 X | 1.2 Gb | 1.2 Gb | 729 | 2439 | 28.9 | 13 | 0.1387 | 1439 | www.ncbi.nlm.nih.gov/datasets/genome/GCA_024433245.1/ | PRI |
|  | Illumina | NO, scaffold | 39.22 X | 1.2 Gb | 1.2 Gb | 728 | 15468 | 28.9 | 13 | 0.1387 | 2438 | www.ncbi.nlm.nih.gov/datasets/genome/GCA_024433255.1/ | ALT |
|  | Illumina | NO, scaffold | 80 X | 1.2 Gb | 1.0 Gb | 13809 | 552476 | 33 | 11 | 0.0025 | 128959 | www.ncbi.nlm.nih.gov/datasets/genome/GCA_035592075.1/ |  |
|  |  |  |  |  |  |  |  |  |  |  |  |  |  |
| *F. peregrinus* | **PacBio HiFi** | **NO, contig** | **125 X** | **1.4 Gb** | **1.4 Gb** | **849** | **849** | **60.8** | **9** | **60.8** | **9** | **www.ncbi.nlm.nih.gov/datasets/genome/GCA_052915955.1/** |  |
|  | PacBio HiFi,  HiC, Bionano DLS |  | 28 X | 1.3 Gb | 1.3 Gb | 124 | 356 | 93.7 | 6 | 15.7 | 24 | www.ncbi.nlm.nih.gov/datasets/genome/GCF_023634155.1/ | PRI |
|  | PacBio HiFi | NO, contig | 28 X | 1.2 Gb | 1.2 Gb | 2745 | 2745 | 1.5 | 235 | 1.5 | 235 | www.ncbi.nlm.nih.gov/datasets/genome/GCA_023634185.1/ | ALT |
|  | PacBio, Arima2 | NO, contig | 72 X | 1.2 Gb | 1.2 Gb | 329 | 705 | 113.5 | 5 | 5.5 | 65 | www.ncbi.nlm.nih.gov/datasets/genome/GCA_965282645.1/ | HAP2 |
|  | PacBio, Arima2 | NO, contig | 72 X | 1.3 Gb | 1.3 Gb | 295 | 728 | 93.4 | 6 | 5.5 | 67 | www.ncbi.nlm.nih.gov/datasets/genome/GCA_965282525.1/ | HAP1 |
|  | Illumina | YES | 137.6 X | 1.1 Gb | 1.1 Gb | 72 | 60351 | 26.8 | 12 | 0.034 | 9833 | www.ncbi.nlm.nih.gov/datasets/genome/GCA_001887755.1/ |  |
|  | Illumina | NO, scaffold | 61 X | 1.2 Gb | 1.2 Gb | 26817 | 49737 | 0.1947 | 1643 | 0.0886 | 3608 | www.ncbi.nlm.nih.gov/datasets/genome/GCA_012488915.1/ |  |
|  | Illumina | NO, scaffold | 41 X | 1.2 Gb | 1.2 Gb | 567 | 15981 | 25.9 | 12 | 0.1357 | 2478 | www.ncbi.nlm.nih.gov/datasets/genome/GCA_024433225.1/ | PRI |
|  | Illumina | NO, scaffold | 41 X | 1.2 Gb | 1.2 Gb | 567 | 15999 | 25.9 | 12 | 0.1353 | 2488 | www.ncbi.nlm.nih.gov/datasets/genome/GCA_024432065.1/ | ALT |
|  | Illumina | NO, scaffold | 61.3 X | 1.2 Gb | 1.2 Gb | 398 | 16096 | 40.6 | 9 | 0.1332 | 2477 | www.ncbi.nlm.nih.gov/datasets/genome/GCA_024431005.1/ | PRI |
|  | Illumina | NO, scaffold | 61.3 X | 1.2 Gb | 1.2 Gb | 398 | 16090 | 40.6 | 9 | 0.1327 | 2478 | www.ncbi.nlm.nih.gov/datasets/genome/GCA_024431015.1/ | ALT |

Supplementary Table 1. Principal genome assembly metrics for *Falco biarmicus* (FBA) and *Falco peregrinus* (FPA) generated in this study (highlighted in bold), compared with publicly available genome assemblies. Reported metrics include sequencing technology, assembly level, genome coverage, assembly size, scaffold and contig statistics (N50 and L50), and corresponding NCBI accession links. PRI: primary assembly; ALT: alternate assembly; HAP: haplotype assembly.

|  | *Falco biarmicus* | *Falco peregrinus* |
| --- | --- | --- |
| Complete BUSCOs | 8,109 (97.3%) | 8,105 (97.2%) |
| Complete and Single copy BUSCOs | 7,945 (95.3%) | 7,193 (94,9%) |
| Complete and Duplicated BUSCOs | 164 (2.0%) | 192 (2.3%) |
| Fragmented BUSCOs | 45 (0.5%) | 44 (0.5%) |
| Missing BUSCOs | 184 (2.2%) | 189 (2.3%) |

Supplemental Table 2. BUSCO completeness assessment of the *Falco biarmicus* and *Falco peregrinus* genome assemblies using the aves_odb10 dataset, reporting complete (single-copy and duplicated), fragmented, and missing conserved orthologs.

| **Assembly** | **QV** | **Error Rate** | **K-mer completeness** |
| --- | --- | --- | --- |
| FPA | 61.04 | 7.87 × 10⁻⁷ | 98.09 |
| FBA | 62.38 | 5.79 × 10⁻⁷ | 96.30 |

Supplemental Table 3. Merqury-based assembly quality metrics for the *Falco biarmicus* (FBA) and *Falco peregrinus* (FPA) genome assemblies, including consensus quality value (QV), estimated base-level error rate, and k-mer completeness.

|  | *Falco biarmicus* | *Falco peregrinus* |
| --- | --- | --- |
| hAT | 158 | 193 |
| CACTA | 3,151 | 3,334 |
| Mutator | 1,246 | 1,242 |
| Tc1-mariner | 187 | 186 |
| PIF-Harbinger | 228 | 242 |
| LTR-RT | 146 | 130 |
| Unknown | 83 | 83 |
| Total | 5,199 | 5,410 |

Supplemental Table 4. Composition of transposable element (TE) libraries generated using EDTA for the *Falco biarmicus* and *Falco peregrinus* genome assemblies, showing the number of elements identified for each major TE superfamily.

|  | *Falco biarmicus* | *Falco peregrinus* | SVs |
| --- | --- | --- | --- |
| hAT | 0.07 | 0.04 | 0.04 |
| CACTA | 1.62 | 1.26 | 1.86 |
| Mutator | 0.42 | 1.65 | 0.90 |
| Tc1-mariner | 0.02 | 0.00 | 0.11 |
| PIF-Harbinger | 0.14 | 0.11 | 0.06 |
| LTR-RT | 1.81 | 2.08 | 35.16 |
| CR1 | 3.35 | 3.30 | 2.94 |
| Total | 7.43 | 8.44 | 41.07 |

Supplemental table 5. Proportion of genome sequence and structural variant (SV) sequence occupied by major transposable element (TE) classes in *Falco biarmicus* and *Falco peregrinus*, highlighting enrichment of specific TE families within SVs relative to genome-wide averages.


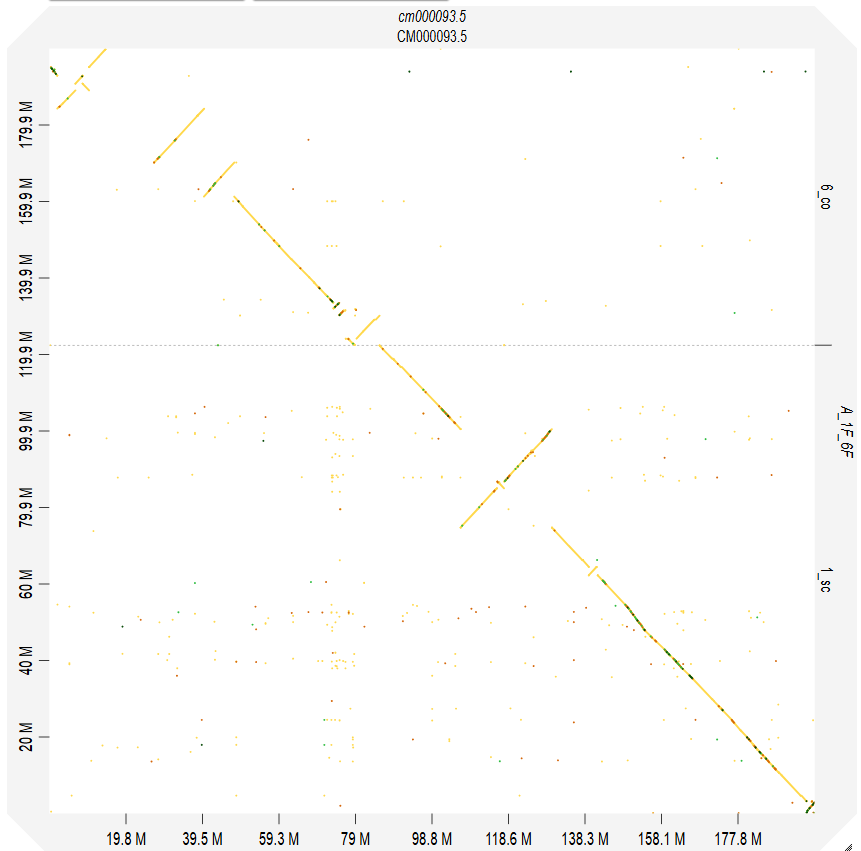


Supplemental Figure 1. Dot plot comparison between *Gallus gallus* chromosome 1 (x-axis) and homologous contigs from *Falco rusticolus* (y-axis), illustrating conserved synteny and chromosomal organization relative to the *Gallus gallus* reference genome.


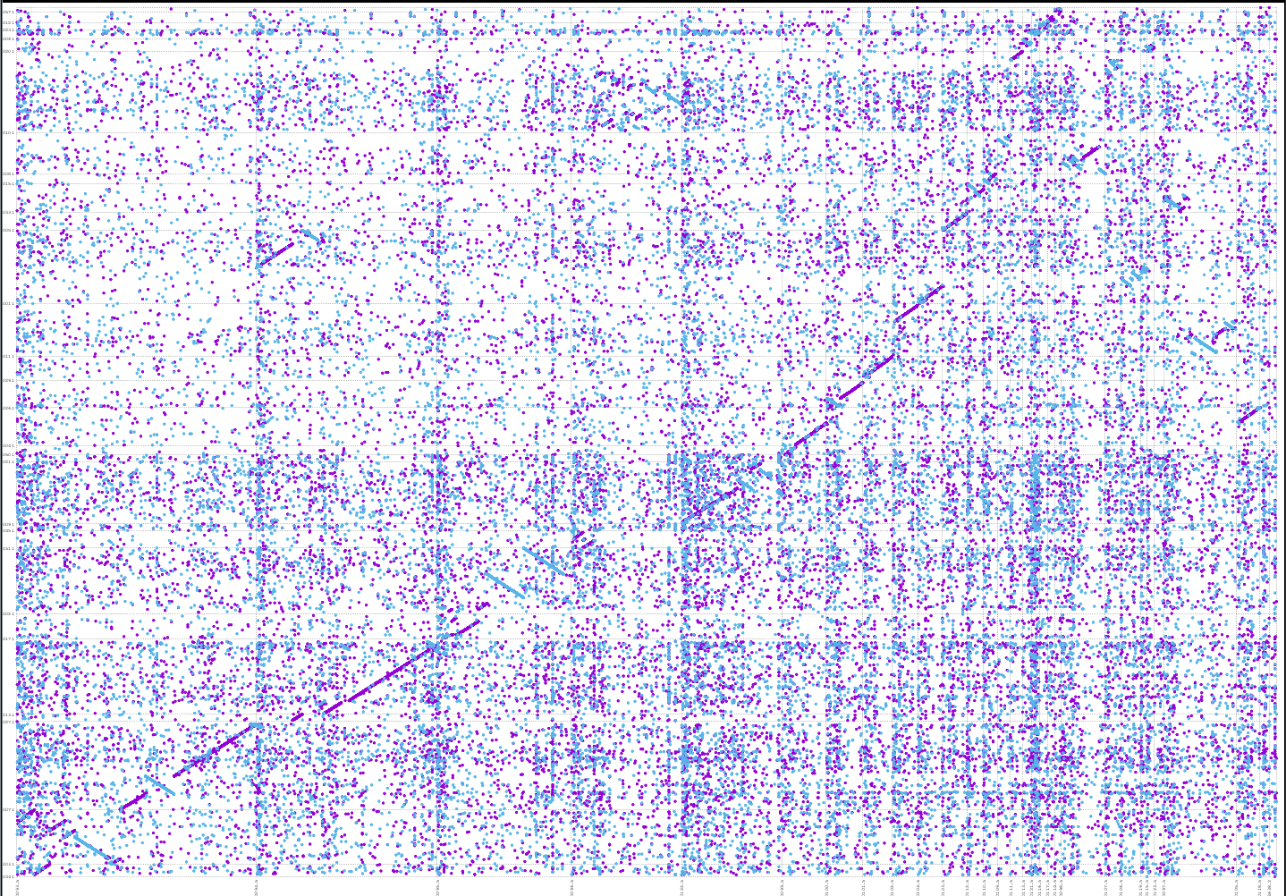


A


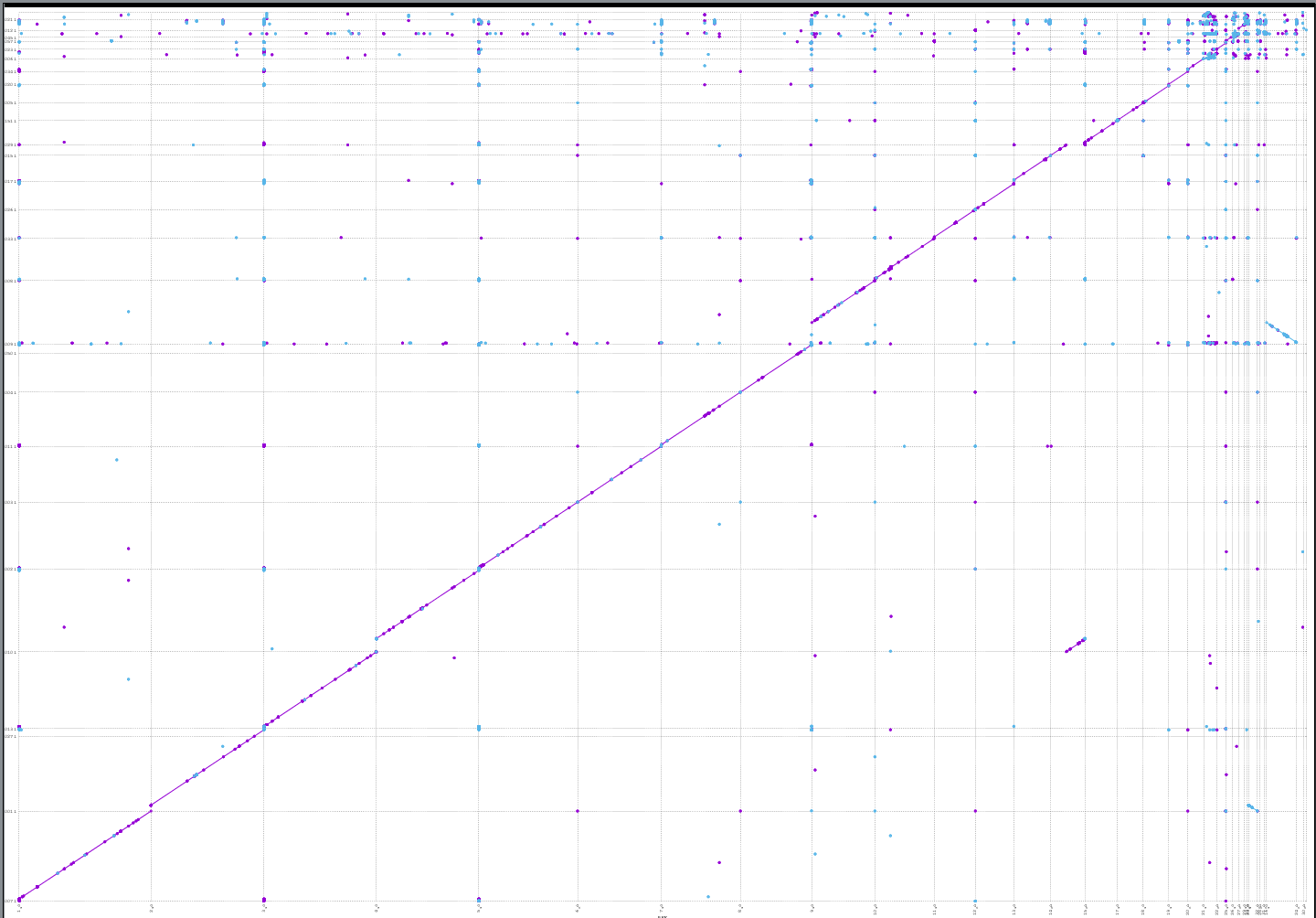


B


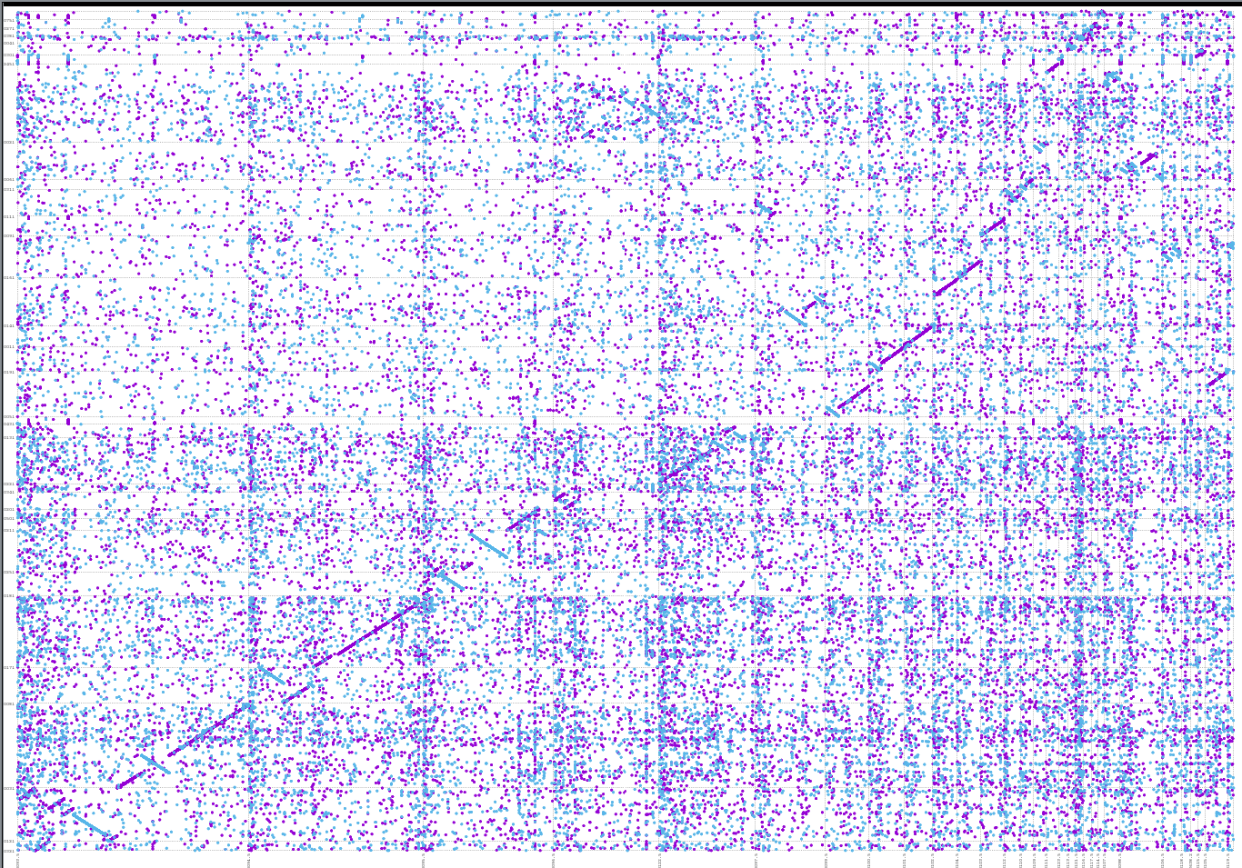


C


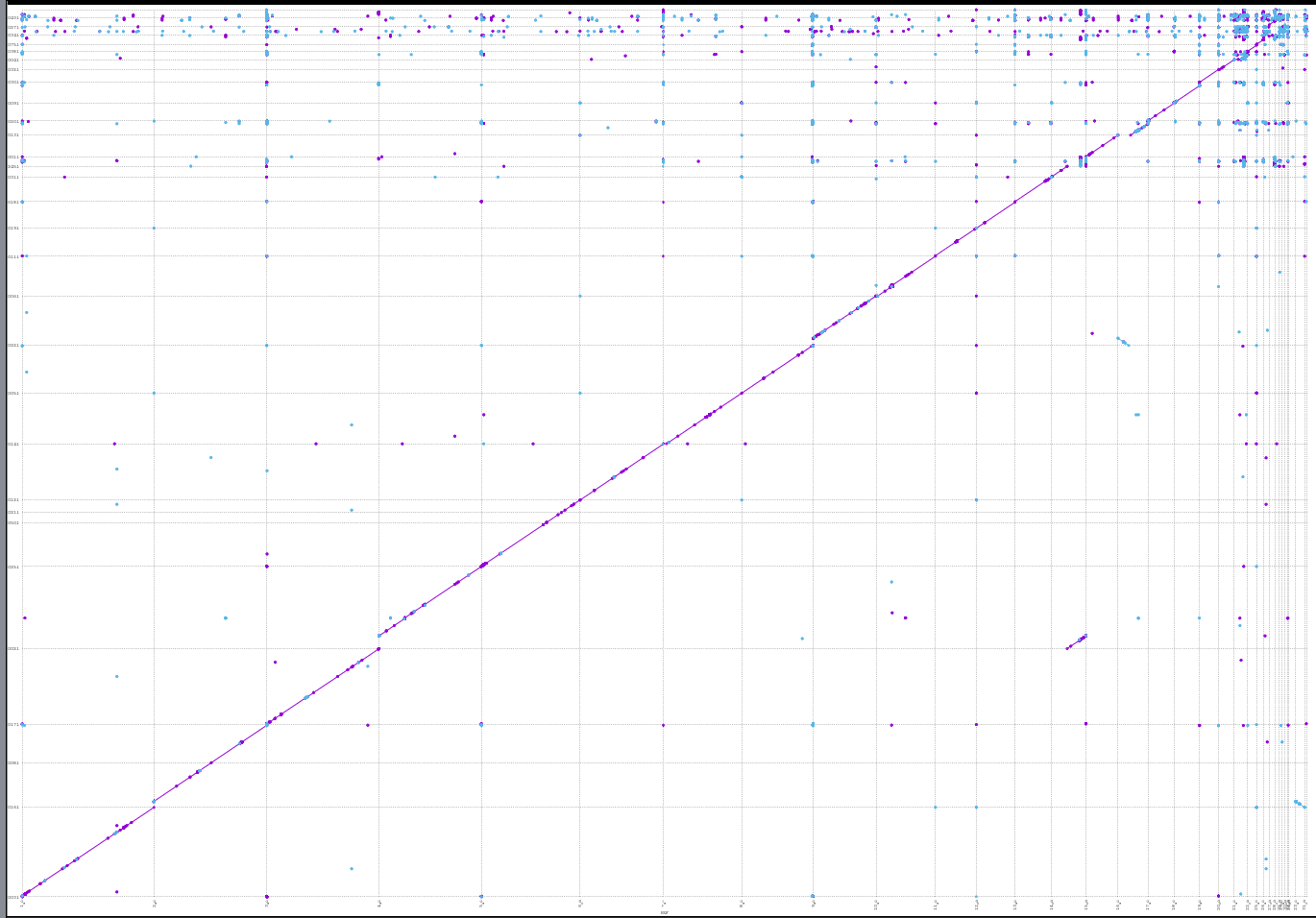


D

Supplemental Figure 2d. Dot plot comparison of *F.rusticolus* (x-axis) and *F. peregrinus* (y-axis)

Pairwise whole-genome dot plot comparisons among *Falco biarmicus*, *Falco peregrinus*, *Falco rusticolus*, and *Gallus gallus*.

(A) *Gallus gallus* (x-axis) versus *Falco biarmicus* (y-axis).

(B) *Falco rusticolus* (x-axis) versus *Falco biarmicus* (y-axis).

(C) *Gallus gallus* (x-axis) versus *Falco peregrinus* (y-axis).

(D) *Falco rusticolus* (x-axis) versus *Falco peregrinus* (y-axis).


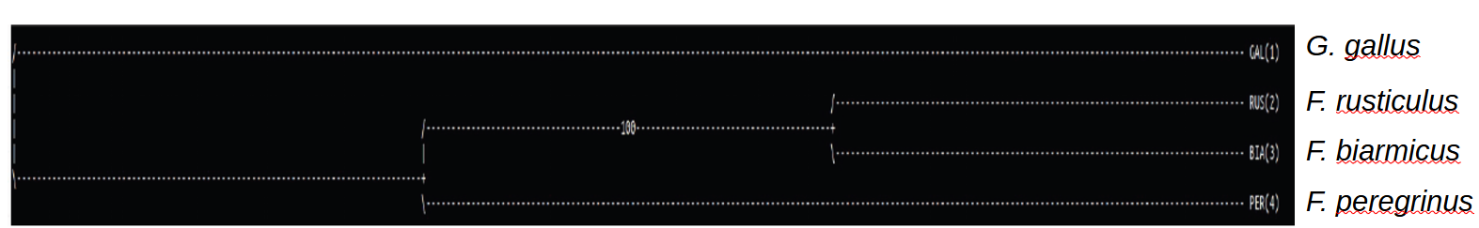


Supplemental figure 3. Neighbor-joining phylogenetic tree based on chromosome 1 sequences from *Falco biarmicus*, *Falco peregrinus*, and *Falco rusticolus*, with *Gallus gallus* used as an outgroup,
